# HLA-haploidentical hematopoietic stem cell transplantation with low-dose thymoglobulin GVHD prophylaxis for an adult T cell leukemia/lymphoma patient treated with pretransplant mogamulizumab

**DOI:** 10.1007/s00277-016-2856-y

**Published:** 2016-10-21

**Authors:** Makoto Hirosawa, Takehiro Higashi, Atsushi Iwashige, Takahiro Yamaguchi, Junichi Tsukada

**Affiliations:** Hematology, University of Occupational and Environmental Health, 1-1 Iseigaoka, Yahatanishi, Kitakyushu, 807-8556 Japan

Dear Editor,

Allogeneic hematopoietic stem cell transplantation (allo-HSCT) has been considered as standard therapy for aggressive adult T cell leukemia/lymphoma (ATLL) patients to achieve a long-term survival [1, 2]. Anti-CCR4 monoclonal antibody mogamulizumab (Mog) is the first immunotherapeutic agent targeting ATLL. Since CCR4 is expressed on regulatory T cells (Treg), there is growing concern regarding increased risk of severe and refractory acute graft-versus-host disease (aGVHD) in ATLL patients treated with Mog before allo-HSCT (pretransplant Mog) [3–6].

We describe a case of an acute-type ATLL patient who underwent HLA-haploidentical peripheral blood stem cell transplantation (haplo-PBSCT) following pretransplant Mog. The interval between the last Mog administration and haplo-PBSCT was 49 days. A 65-year-old Japanese male presented with a 2-week history of fatigue, anorexia, and nausea. He was diagnosed as acute-type ATLL with hypercalcemia. His ATLL including abnormal bone lesions was chemorefractory. Therefore, he was further treated with two doses of Mog (1 mg/kg weekly for 2 weeks). The disease status significantly improved after Mog administration. Unmanipulated haplo-PBSCT was performed from his son (Table 1). The conditioning consisted of fludarabine (25 mg/m^2^/day, days −8 to −4), melphalan (80 mg/m^2^, day −3), rabbit anti-thymocyte globulin (thymoglobulin; 2.5 mg/kg, day −2), and TBI (4 Gy). Tacrolimus and mycophenolate mofetil were further used for GVHD prophylaxis. Stable and complete donor chimerism was obtained. On day 30, he only developed grade I aGVHD (stage 1 skin rash). One year later, the number of CD4 + CD25 + CD127-/low Treg cells and CD4 + T cells in his PB remained below the normal range (15/μL and 183/μL, respectively), showing delayed T cell reconstitution. He has been in complete remission (CR) without chronic GVHD for 2 years.

**Table 1:** HLA status of the patient and donor

	Sex	Relation	Blood type	HLA-A	HLA-B	HLA-C	HLA-DR
Recipient	Male		A+	0201/2402	3901/5502	0702/0102	1201/0901
Donor	Male	Son	A+	0201/0206	3901/5101	0702/1402	1201/0803

Low-dose thymoglobulin, in addition to its T cell depleting properties, stimulates the recovery of Treg cells [7, 8]. In this regard, Motohashi et al. reported an acute-type ATLL patient who achieved CR following Mog treatment and subsequently received unrelated bone marrow transplantation (uBMT) [9]. Two months after the last Mog administration, uBMT was performed from a serologically two antigen (HLA-C and -DR) mismatched donor by using conditioning with 1.25 mg/kg of thymoglobulin. As a result, they only observed grade I aGVHD.

In ATLL, allo-HSCT at disease regression induced by initial treatment has been shown to improve the clinical outcome [10]. Pretransplant Mog significantly improved the disease status before haplo-PBSCT. Moreover, a recent nationwide study of ATLL patients with pretransplant Mog in Japan showed that pretransplant Mog with intervals of <50 days to allo-HSCT was associated with increased risk of GVHD-related mortality [6]. In our case, the interval between pretransplant Mog and haplo-PBSCT was 49 days. The nationwide study also pointed out the possibility that in vivo effector T cell depletion with anti-thymocyte globulin contributes to the reduction of severe aGVHD in ATLL patients with pretransplant Mog [6]. To our knowledge, our case is the first report of successful prevention of aGVHD with low-dose thymoglobulin in haplo-PBSCT following pretransplant Mog. Low-dose thymoglobulin is an effective option for aGVHD prophylaxis in ATLL patients with pretransplant Mog.
